# Exosomal HSP90 induced by remote ischemic preconditioning alleviates myocardial ischemia/reperfusion injury by inhibiting complement activation and inflammation

**DOI:** 10.1186/s12872-023-03043-y

**Published:** 2023-02-01

**Authors:** Xiao-Fang Cheng, Shi-Tao He, Guo-Qiang Zhong, Jian-Jun Meng, Min Wang, Qi Bi, Rong-Hui Tu

**Affiliations:** 1 Department of Cardiology, First Affiliated Hospital, Guangxi Medical University, Nanning, 530021 Guangxi China; 2 Geriatric Healthcare Center, First Affiliated Hospital, Guangxi Medical University, Nanning, 530021 Guangxi China; 3 Guang Xi Key Laboratory of Precision Medicine in Cardio-Cerebrovascular Disease Control and Prevention, Nanning, 530021 Guangxi China; 4 Guang Xi Clinical Research Center for Cardio-Cerebrovascular Diseases, Nanning, 530021 Guangxi China; 5 Department of Geriatric Cardiology, First Affiliated Hospital, Guangxi Medical University, 6 Shuangyong Road, Nanning, 530021 Guangxi China

**Keywords:** Remote ischemic preconditioning, Exosomes, HSP90, Complement system, Inflammation

## Abstract

**Background/Aims:**

The activation of the complement system and subsequent inflammatory responses are important features of myocardial ischemia/reperfusion (I/R) injury. Exosomes are nanoscale extracellular vesicles that play a significant role in remote ischemic preconditioning (RIPC) cardioprotection. The present study aimed to test whether RIPC-induced plasma exosomes (RIPC-Exo) exert protective effects on myocardial I/R injury by inhibiting complement activation and inflammation and whether exosomal heat shock protein 90 (HSP90) mediates these effects.

**Methods:**

Rat hearts underwent 30 min of coronary ligation followed by 2 h of reperfusion. Plasma exosomes were isolated from RIPC rats and injected into the infarcted myocardium immediately after ligation. Sixty rats were randomly divided into Sham, I/R, I/R + RIPC-Exo (50 µg/µl), and RIPC-Exo + GA (geldanamycin, 1 mg/kg, administration 30 min before ligation) groups. Cardiomyocyte apoptosis, the release of myocardial markers (LDH, cTnI and CK-MB), infarct size, the expression of HSP90, complement component (C)3, C5a, c-Jun N-terminal kinase (JNK), interleukin (IL)-1β, tumor necrosis factor (TNF)-alpha and intercellular adhesion molecule -1 (ICAM-1) were assessed.

**Results:**

RIPC-Exo treatment significantly reduced I/R-induced cardiomyocyte apoptosis, the release of myocardial markers (LDH, cTnI and CK-MB) and infarct size. These beneficial effects were accompanied by decreased C3 and C5a expression, decreased inflammatory factor levels (IL-1β, TNF-α, and ICAM-1), decreased JNK and Bax, and increased Bcl-2 expression. Meanwhile, the expression of HSP90 in the exosomes from rat plasma increased significantly after RIPC. However, treatment with HSP90 inhibitor GA significantly reversed the cardioprotection of RIPC-Exo, as well as activated complement component, JNK signalling and inflammation, indicating that HSP90 in exosomes isolated from the RIPC was important in mediating the cardioprotective effects during I/R.

**Conclusion:**

Exosomal HSP90 induced by RIPC played a significant role in cardioprotection against I/R injury, and its function was in part linked to the inhibition of the complement system, JNK signalling and local and systemic inflammation, ultimately alleviating I/R-induced myocardial injury and apoptosis by the upregulation of Bcl-2 expression and the downregulation of proapoptotic Bax.

**Supplementary Information:**

The online version contains supplementary material available at 10.1186/s12872-023-03043-y.

## Introduction

Timely recovery of coronary flow and then successful restoration of myocardial perfusion is the best strategy to salvage the myocardium and significantly improve clinical outcomes following acute myocardial infarction. However, reperfusion itself may induce a systemic inflammatory response and even fatal cardiac dysfunction by so-called ischemic/reperfusion (I/R) injury. Developing effective strategies for alleviating I/R injury remains challenging [1, 2].

Remote ischemic preconditioning (RIPC) is also known as intermittent I/R intervening in the leg or distant organ before myocardial ischemia, and it has been proven to relieve myocardial I/R injury in various models [3–6].

The protective mechanisms of this novel strategy have been extensively investigated, and emerging data provide evidence that circulating extracellular vesicles released during RIPC are critical for its cardioprotection against I/R injury [7–10]. Currently, the great therapeutic potential of extracellular vesicles for cardiovascular diseases has received increasing attention.

Exosomes are nanoscale extracellular vesicles (30–150 nm) that widely exist in blood, urine, saliva and cerebrospinal fluid [11]. Exosomes are spontaneously released by a wide range of mammalian cell types and participate in intercellular communication by transmitting a series of signalling molecules, including lipids, proteins, messenger RNAs (mRNAs) and microRNAs [12]. In the past decade, exosomes secreted from different types of stem cells have been proven to play a protective role in myocardial I/R injury [13–15]. Most recently, an increasing number of studies have demonstrated that RIPC attenuates myocardial I/R injury by inducing exosome secretion [10, 16, 17]. However, the detailed protective mechanisms of RIPC-induced exosomes (RIPC-Exo) against myocardial I/R injury have not yet been fully elucidated.

Heat shock protein 90 (HSP90) is the most common and conserved molecular chaperone that plays an important role in protein synthesis, folding, assembly and trafficking [18]. Being activated by ischemia/hypoxia and cellular stress, HSP90 is an emerging therapeutic target in I/R injury and preconditioning cardioprotection [19, 20]. Our laboratory recently revealed that HSP90 is crucial for postconditioning-induced cardioprotection [21–23] and that its activity is linked to the suppression of the complement system, the c-Jun N-terminal kinase (JNK) pathway and inflammatory response [24]. However, whether RIPC-Exo could inhibit complement activation and inflammation after I/R through targeting HSP90 remains unknown.

Complement activation plays a critical role in myocardial I/R injury [25–27]. The complement system is a protein response system that is activated under ischemic and hypoxic conditions [27]. Once activated, it initiates a series of inflammatory responses involving the recruitment of inflammatory cells and the release of inflammatory cytokines, including interleukin (IL) 1β, tumor necrosis factorα (TNF-α) and intercellular adhesion molecule1 (ICAM-1), which contribute to maladaptive myocardial I/R injury [28, 29]. In fact, the excessive activation of the complement products C3 and C5a are worsened factors for myocardial necrosis during I/R [30]. Moreover, the production of the anaphylatoxins C3 and C5a can trigger the JNK pathway to aggravate inflammatory reactions [31]. Hence, complement/JNK signalling displays promise as a therapeutic target for I/R injury. However, it is not known whether RIPC-Exo is involved in I/R injury via the complement/JNK signalling pathway.

In the present study, we aimed to investigate the protective effect of RIPC-Exo on myocardial I/R injury in rats and the underlying mechanism. We propose the hypothesis that RIPC-Exo may transport HSP90 to cardiomyocytes to protect against I/R injury by decreasing myocardial apoptosis via the inhibition of complement activation, inflammation and JNK signalling. This study provides an interesting therapeutic strategy for the treatment of I/R injury.

## Materials and methods

### Animals

Pathogen-free Sprague Dawley rats (similar body weight 250 ± 20 g) were provided by the Experimental Animal Centre of Guangxi Medical University, China. Rats were kept in cages under normal laboratory conditions (temperature: 24 °C; photoperiod: 12 h light–12 h dark). Food and water were freely available to the rats. All animal experiments were conducted according to protocols authorised by the Institutional Animal Care and Use Ethics Committee.

### RIPC and myocardial I/R models

After the rats were sedated with 5% sodium pentobarbital (45 mg/kg), RIPC was performed by tying both lower extremities (middle–upper 1/3) with elastic rubber bands for 5 min, then relaxing them for five minutes and repeating this procedure four times [9, 10]. To create a model of myocardial I/R injury, rats were ventilated with a small animal positive pressure ventilator and electrocardiogram leads were placed to monitor heart rate. An intercostal incision was made between the fourth and fifth cords of rats for access to the thorax. Using the slipknot method, the left anterior descending (LAD) coronary artery was ligated with 6–0 silk sutures and a small plastic tube was inserted through the ligature to form a snare for reversible LAD occlusion. Myocardial ischemia was induced by compressing the LAD by tightening the ligature around the plastic tube and confirmed by the bleached appearance of the ligated segments and elevation of the ST segment. After 30 min of ischemia, the ligature was loosened to reperfuse for 2 h. At the end of reperfusion, the rats were euthanized. Myocardial tissue from the left ventricle and serum samples were collected for future investigation.

### Experimental protocol

The rats were randomly divided into two groups (n = 5) to obtain plasma exosomes. (1) Control Exo (CON-Exo) group: no operation; (2) RIPC-Exo group: RIPC operation was performed. For the in vivo experiment, the rats were randomly divided into four groups (n = 15). (1) Sham group: normal rats were injected with 10 μL PBS and not ligated for 150 min; (2) I/R group: I/R rats were injected with PBS (10 μL PBS, intramuscular injection, after the induction of ischaemia); (3) I/R + RIPC-Exo group: I/R rats were injected with RIPC-Exo (10 μL PBS[≈ 50 μg RIPC-Exo] intramyocardial injection, after the induction of ischaemia); (4) RIPC-Exo + GA group: I/R + RIPC-Exo rats were injected with GA (GA, 1 mg/kg, intraperitoneally, 30 min before ischemia).

### Plasma exosome extraction

Laparotomy was performed in the control and RIPC groups, and blood was collected from the abdominal aorta with a 7# needle and immediately removed into K2-EDTA tubes. The blood samples were centrifuged at 2000 g for 15 min to collect the plasma. Exosomes were isolated from the plasma of the control and RIPC groups. Exosomes were formulated as follows: differential ultracentrifugation (UC) at 4 °C. Samples were centrifuged at 2000 g for 15 min to obtain plasma, and then at 10,000 g for 30 min, the supernatants were collected and centrifuged at 100,000 g for 90 min. Following a series of differential centrifugation steps, the final precipitation was categorised as exosomes, resuspended in 100 μl of PBS and stored at -80 °C for subsequent experiments.

### Exosome identification

Exosomes were observed using transmission electron microscopy (TEM). The exosome solution was placed on a 200-mesh copper electron microscopy grid coated with Formvar/carbon and incubated for 1 min at 25 deg. The exosomes were then incubated for a further minute at 25 °C under three percent phosphotungstic acid staining. The exosomes were then examined under a transmission electron microscope. The diameter of the exosomes was measured using photomicrographs. Nanoparticle tracking analysis (NTA) was performed using Zeta View (Particle Metrix) to determine the size distribution and concentration of the exosomes. Western blotting was used to detect exosomal markers (CD63, TSG101).

### Measurement of myocardial infarct size

To determine infarct size, experiments were performed in the four groups. After two hours of reperfusion, the LAD coronary artery was ligated, and the inferior vena cava was injected with 2% Evans blue. Stained hearts were frozen, cut horizontally into 1–2 mm thicknesses and treated with 2,3,5-triphenyl tetrazolium chloride (1%; TTC, Sigma-Aldrich) for 15 min at 37 °C to determine myocardial infarct size. The total left ventricular and infarct size areas were calculated with Image-Pro Plus 6.0 (Media101 Cybernetics, Bethesda, MD) after fixation in 10% formaldehyde.

### Levels of cTnI, CK-MB and LDH in serum

After reperfusion, blood samples were collected in a 5-mL vacuum tube and centrifuged at 2000 g for 10 min. The supernatant was stored at -80 °C. According to the manufacturer’s instructions, cTnI values were measured using a cTnI enzyme-linked immunosorbent assay kit (ELISA, CUSABIO, China) on a 7600 automated analyser (Hitachi, Tokyo, Japan). The CK-MB and LDH levels were evaluated using an automated biochemical analyser (IDEXX Laboratories Inc., Maine, USA).

### Levels of inflammatory factors in the serum

Blood concentrations of IL-1β, TNF-α and ICAM-1 were determined using ELISA kits according to the manufacturer’s instructions (CUSABIO, Wuhan, China). Absorbance was measured with a spectrophotometer, and the results were expressed in a μg/ml serum.

### TUNEL assay for myocardial apoptosis

Tissues were dehydrated and embedded after being fixed in 4% paraformaldehyde. The TUNEL kit was used to stain the cells (Roche Diagnostics, Basel, Switzerland). Apoptosis was then detected with positive fluorescence microscopy. Five randomly selected image fields were acquired for each segment, and the ratio of apoptotic cells to total cardiomyocytes (apoptotic index, %) was calculated using Image J software.

### RNA isolation and quantitative real-time polymerase chain reaction

Total RNA was extracted with TRIzol reagent (Invitrogen, USA), and the obtained RNA was reverse transcribed using the MonScript Reverse Transcription Kit (Monad, China). The polymerase chain reaction was performed on a 7500 real-time fluorescence quantitative PCR instrument using the SYBR® Green qPCR Mix Kit (Monad, China). GAPDH served as the control gene. The corresponding primers used in this study were IL-1β forward 5′-AATCTCACAGCAGCATCTCGACAAG-3′ and reverse 5′-TCCACGGGCAAGACATAGGTAGC-3′, TNF-α forward 5′-ATGGGCTCCCTCTCATCAGTTCC-3′ and reverse 5′-CCTCCGCTTGGTGGTTTGCTAC-3′, and ICAM-1 forward 5′-TGTCGGTGCTCAGGTATCCATCC-3′ and reverse 5′-TTCGCAAGAGGAAGAGCAGTTCA-3′. The 2^−ΔΔCt^ approach was used to study expression.

### Western blot analysis

First, RIPA lysate (Beyotime, Shanghai, China) was used to extract the total amount of protein from exosomes and tissues. The protein concentration was then determined using a Bicinchoninic Acid (BCA) Protein Assay Kit. After electrophoresis on a 10% sodium dodecyl sulphate–polyacrylamide gel, the samples were transferred to a polyvinylidene difluoride (PVDF) membrane. Full-length membranes were blocked in TBST containing 5% skim milk and cut into strips based on the molecular weight of the target protein and then incubated overnight at 4 °C with appropriate primary antibodies CD63 (Santa Cruz, TX, USA), TSG101 (Abcam, Cambridge, UK), HSP90 (Proteintech, Wuhan, China), Bax (Proteintech, Wuhan, China), Bcl-2 (Signalway Antibody, Baltimore, MD), C3 (Abcam, Cambridge, UK), C5a (Invitrogen, USA) and JNK (Abcam, Cambridge, UK). Horseradish peroxidase-labelled goat antirabbit and rabbit antimouse antibodies (Proteintech, Wuhan, China) were incubated at room temperature for one hour. A fluorescent chemical FC3 (ProteinSimple, Santa Clara, CA, USA) imaging device was used to detect the signals, while protein expression was analysed using ImageJ software (Additional file 1).

### Statistical analysis

SPSS 25.0 statistical analysis software was used to analyse the data. Measured data were presented as mean ± standard deviation ($\overline{x}\pm \mathrm{s }$). The two groups were compared using the Student’s t-test, and one-way analysis of variance (ANOVA) was used to evaluate the means of the multiple groups. Statistical significance was defined as *P* < 0.05.

## Results

### RIPC promotes exosome release

**Fig. 1:**
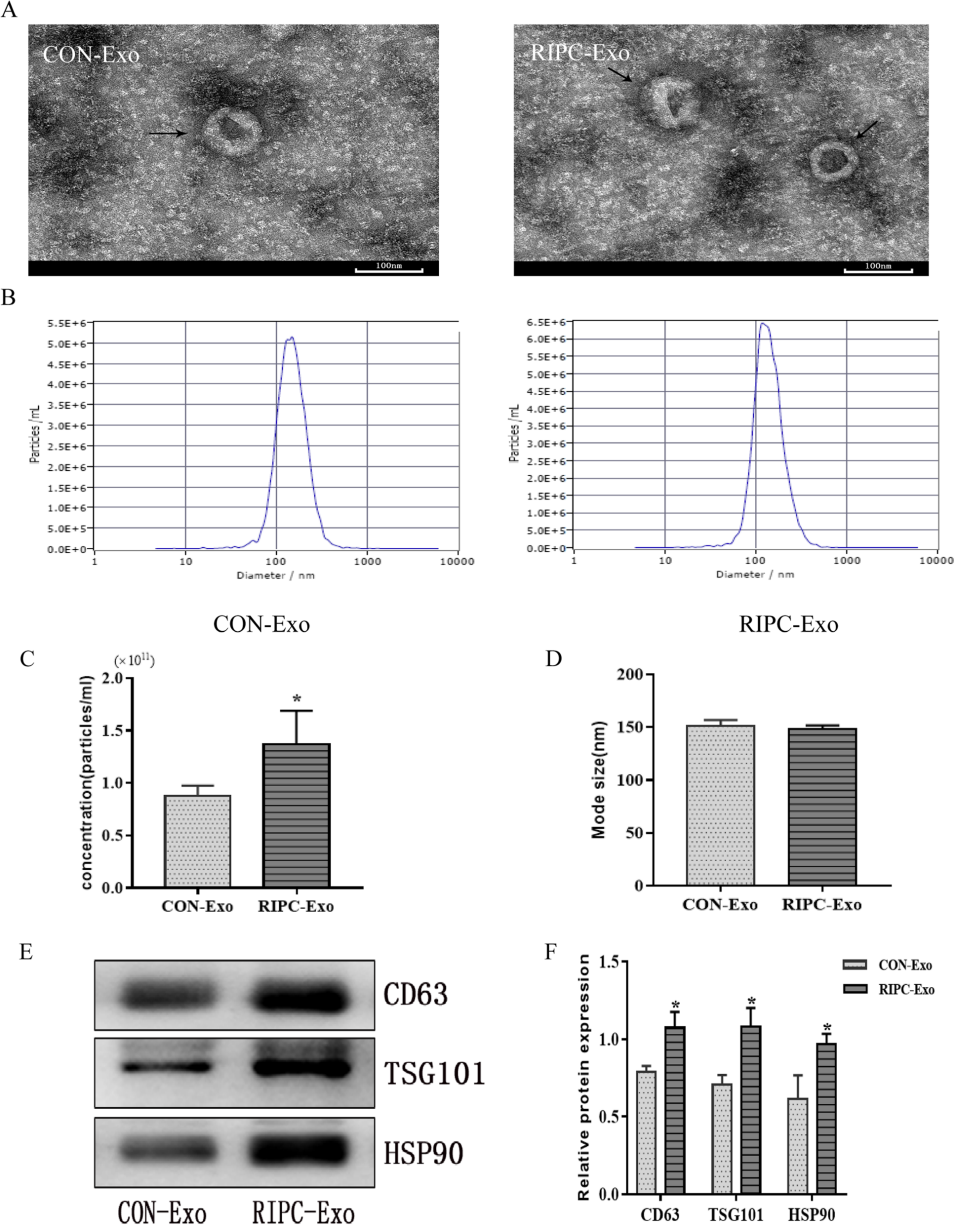
Characterization of plasma exosomes. **A** Representative results of the transmission electron microscope (TEM) demonstrate the morphology and size of exosomes. **B** Representative results of nanoparticle tracking analysis (NTA) demonstrate similar size distribution of exosomes in RIPC and control groups. **C**–**D** Average concentration and size of exosomes in control exosomes and RIPC exosomes. **E** Representative Western blots showing the expression of CD63, TSG101 and HSP90. **F** Relative expression of CD63, TSG101 and HSP90. The results are represented by mean ± standard deviation $\stackrel{-}{(x}\pm s$). *Ρ < 0.05 versus CON-Exo; n = 3 for each group

Exosomes were isolated from the plasma of the control and RIPC rats according to a standard UC protocol. TEM analysis revealed that the particles in both groups of rats were typical ‘cup-shaped’ membrane-bound vesicles with a diameter of ~ 100 nm (Fig. 1A). Nanoparticle tracking analysis (NTA) also showed the size range and concentration of the particles (Fig. 1B). The concentration of the particles was significant differences between RIPC-Exo and CON-Exo (1.50 ± 0.50 × 10^11^ vs. 8.80 ± 0.95 × 10^10^ particles/ml, *Ρ* < 0.05), and the distribution of the diameter of most particles was between 50 and 200 nm, with an average value of 140 ± 4.30 nm, *P* > 0.05 (Fig. 1C, D). In addition, Western blotting identified the expression of exosomal markers, such as CD63 and TSG101 (Fig. 1E), in exosomes, and the levels of these markers were higher in RIPC-Exo than in CON-Exo (*Ρ* < 0.05, Fig. 1F). These results prove that the isolated microvesicles from the plasma of control and RIPC rats are exosomes and that RIPC releases a large number of exosomes.

#### Figure 1

(See Fig. 1).

### Expression of HSP90 in RIPC-Exo

To determine whether HSP90 expression is increased in RIPC-induced exosomes, changes in the protein expression of HSP90 were detected in RIPC exosomes. As shown in Fig. 1E, F, HSP90 was significantly increased in RIPC-Exo (*Ρ* < 0.05).

### Expression of exosomal HSP90 in the I/R model

**Fig. 2:**
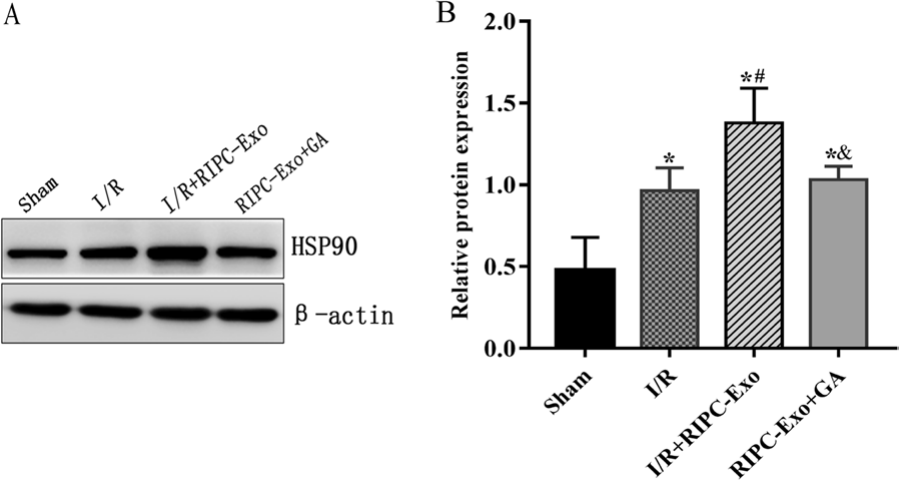
The effects of RIPC-Exo on HSP90 protein expression. **A** Representative Western blots showing the expression of HSP90. **B** Relative expression of HSP90 protein. The results are represented by $\overline{x }\pm s$. *Ρ < 0.05 versus Sham group; ^#^*Ρ* < 0.05 versus I/R group; ^&^*Ρ* < 0.05 versus I/R + RIPC-Exo group; n = 5 for each group

The protein expression of HSP90 in myocardial tissue was identified. Figure 2 shows that the protein concentration of HSP90 was significantly increased in the I/R + RIPC-Exo group compared with the I/R group (*Ρ* < 0.05). Inhibition of HSP90 function with the selective HSP90 inhibitor (GA) prevented the increased expression of HSP90 (*Ρ* < 0.05).

#### Figure 2

(See Fig. 2).

### HSP90 in RIPC-Exo ameliorated myocardial infarct size

**Fig. 3:**
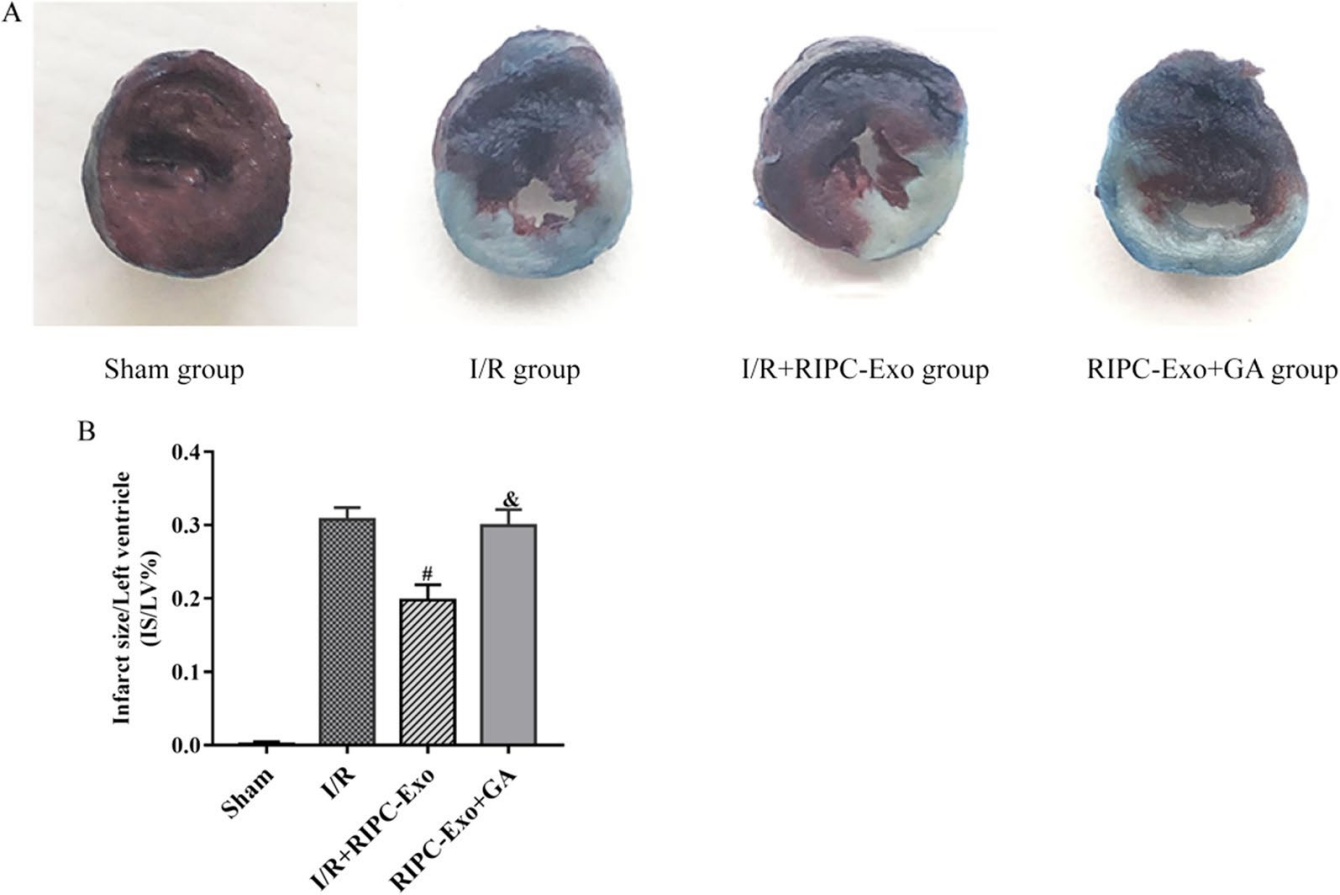
Effects of RIPC-Exo and GA on myocardial infarct size after cardiac I/R injury. **A** Representative images of TTC-EB staining of hearts from different groups. **B** Quantitative analysis of the myocardial infarct size. The results are represented by $\overline{\mathrm{x}}\pm \mathrm{s }$. ^#^*Ρ* < 0.05 versus I/R group; ^&^*Ρ* < 0.05 versus I/R + RIPC-Exo group; n = 5 for each group

As shown in Fig. 3, no myocardial infarction occurred in the sham group. When comparing the I/R + RIPC-Exo group with the I/R group, the infarct size was smaller (19.76 ± 2.09% vs. 30.75 ± 1.65%, *Ρ* < 0.05). However, compared with the I/R + RIPC-Exo group, the infarct size increased significantly in the RIPC-Exo + GA group (19.76 ± 2.09% vs. 29.90 ± 2.20% *Ρ* < 0.05). Thus, GA antagonised the infarct-reducing effect of HSP90 derived from RIPC-Exo.

#### Figure 3

(See Fig. 3).

### HSP90 in RIPC-Exo reduces myocardial injury

This study also examined cardiac serum enzymes, such as cTnI, CK-MB and LDH, which are common markers of myocardial injury. CK-MB (2102.2 ± 125.3 vs. 776.6 ± 131.9; *Ρ* < 0.05), LDH (1433.0 ± 149.8 vs. 487.4 ± 120.2; *Ρ* < 0.05) and cTnI (2.17 ± 0.47 vs. 0.03 ± 0.02; *Ρ* < 0.05) were significantly increased in the I/R group compared with the Sham group. The I/R + RIPC-Exo group had significantly lower levels of CK-MB (1360.6 ± 85.1 vs. 2102.2 ± 125.3; *Ρ* < 0.05), LDH (980.0 ± 160.4 vs. 1433.0 ± 149.8; *Ρ* < 0.05) and cTnI (0.82 ± 0.16 vs. 2.17 ± 0.47; *Ρ* < 0.05) than the I/R group, while GA reversed the effects of RIPC-Exo, suggesting that HSP90 derived from RIPC-Exo inhibited I/R-induced myocardial injury (Table 1).

**Table 1:** Levels of CK-MB, cTnI and LDH in serum

Groups	CK-MB (ng/mL)	cTnI (pg/mL)	LDH (mU/mL)
Sham	776.6 ± 131.9	0.03 ± 0.02	487.4 ± 120.2
I/R	2102.2 ± 125.3*	2.17 ± 0.47*	1433.0 ± 149.8*
I/R + RIPC-Exo	1360.6 ± 85.1*^#^	0.82 ± 0.16*^#^	980.0 ± 160.4*^#^
RIPC-Exo + GA	2090.4 ± 95.9*^&^	1.86 ± 0.42*^&^	1522.6 ± 142.6*^&^

Results are represented by $\overline{\mathrm{x}}\pm \mathrm{s }$. *Ρ < 0.05 versus Sham group; ^#^*Ρ* < 0.05 versus I/R group; ^&^*Ρ* < 0.05 versus I/R + RIPC-Exo group; n = 5 for each group

#### Table 1

(See Table 1).

### HSP90 in RIPC-Exo reduces myocardial apoptosis

**Fig. 4:**
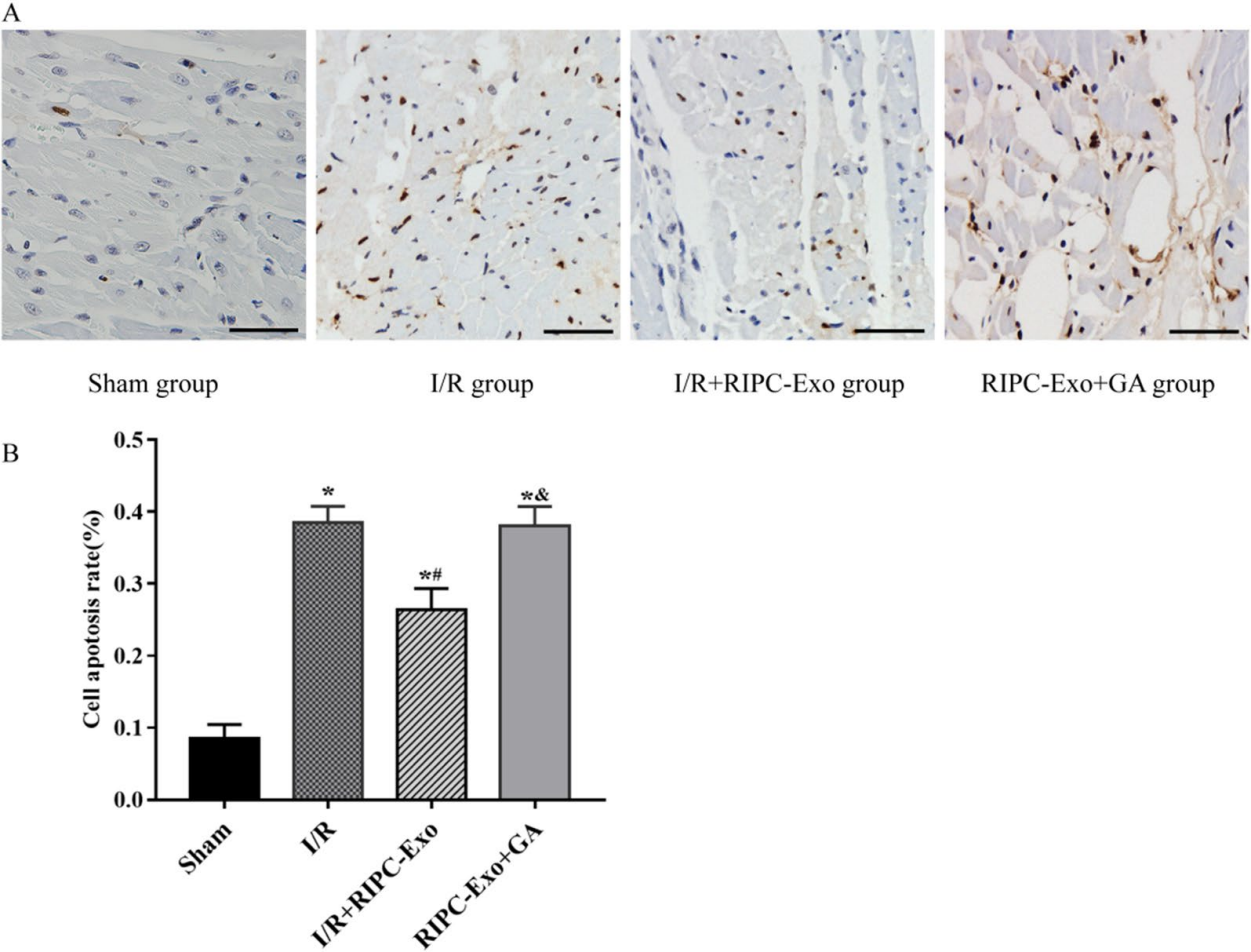
Effects of RIPC-Exo and GA on apoptosis after cardiac I/R injury. **A** Representative images of TUNEL staining of hearts from the different rat groups. **B** Quantitative analysis of TUNEL-positive cardiomyocytes. TUNEL stain × 400, all bars = 20 μm. The results are represented by $\overline{x}\pm \mathrm{s }$. *Ρ < 0.05 versus Sham group; ^#^*Ρ* < 0.05 versus I/R group; ^&^*Ρ* < 0.05 versus I/R + RIPC-Exo group; n = 5 for each group

The TUNEL assay was used to detect myocardial apoptosis. The rate of cardiomyocyte apoptosis was significantly higher in the I/R group than in the Sham group (38.51 ± 2.26% vs. 8.54 ± 1.91%, *Ρ* < 0.05). The rate of cardiomyocyte apoptosis was significantly lower in the I/R + RIPC-Exo group than in the I/R and RIPC-Exo + GA groups (26.44 ± 2.94% vs. 38.51 ± 2.26% and 38.09 ± 2.63%, *Ρ* < 0.05). There was no statistically significant difference between the RIPC-Exo + GA and I/R groups (38.09 ± 2.63% vs. 38.51 ± 2.26%, *Ρ* > 0.05), indicating that GA attenuates the apoptosis inhibitory effects of HSP90 derived from RIPC-Exo (Fig. 4).

#### Figure 4

(See Fig. 4).

### HSP90 in RIPC-Exo reduces the levels of inflammatory factors

**Fig. 5:**
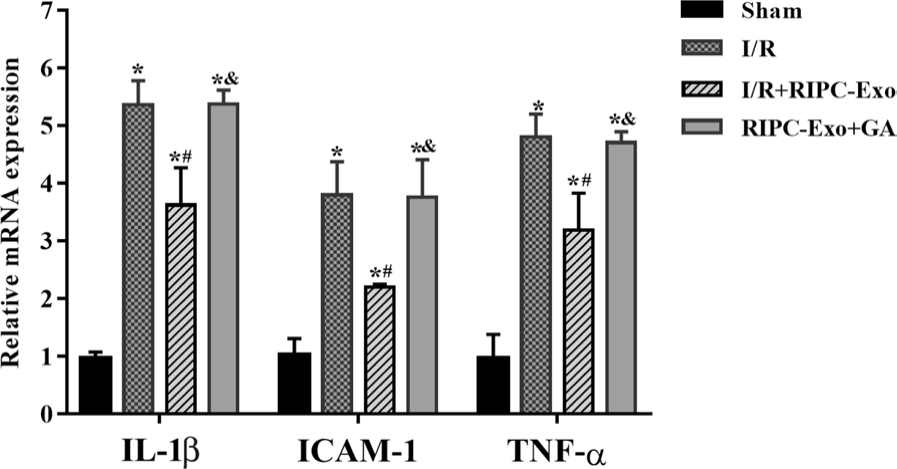
Effects of RIPC-Exo and GA on IL -1β, ICAM-1, and TNF-α mRNA expression. RT-qPCR was used to measure the mRNA expression levels of TNF-α, IL-1β, and ICAM-1 in various groups. Dates are represented by $\overline{x}\pm \mathrm{s }$. *Ρ < 0.05 versus Sham group; ^#^*Ρ* < 0.05 versus I/R group; ^&^*Ρ* < 0.05 versus I/R + RIPC-Exo group; n = 5 for each group

We evaluated the serum and mRNA levels of IL-1β, ICAM-1 and TNF-α expression. Compared with the Sham and I/R + RIPC-Exo groups, the I/R group had increased serum levels of IL-1β (54.04 ± 4.10 vs. 29.70 ± 2.68 and 42.49 ± 3.05, *Ρ* < 0.05), ICAM-1 (57.03 ± 7.19 vs. 26.00 ± 4.28 and 40.83 ± 3.58, *Ρ* < 0.05) and TNF-α (517.13 ± 34.51 vs. 209.96 ± 18.77 and 399.85 ± 27.41, *Ρ* < 0.05). The RIPC-Exo + GA group had higher serum levels of IL-1β (51.86 ± 2.19 vs. 42.49 ± 3.05, *Ρ* < 0.05), ICAM-1 (53.07 ± 6.80 vs. 40.83 ± 3.58, *Ρ* < 0.05) and TNF-α (502.65 ± 23.67 vs. 399.85 ± 27.41, *Ρ* < 0.05) than the I/R + RIPC-Exo group. The I/R and RIPC-Exo + GA groups had identical levels (Table 2). Similar to serum, IL-1β, ICAM-1 and TNF-α mRNA levels were significantly lower in the sham and I/R + RIPC-Exo groups than in the I/R group (*Ρ* < 0.05, Fig. 5). Likewise, GA therapy reversed this effect (*Ρ* < 0.05). HSP90 derived from RIPC-Exo seemed to reduce the inflammation of the myocardium induced by I/R.

**Table 2:** Levels of IL-1β, ICAM-1 and TNF-α in serum

Groups	IL-1β(pg/mL)	ICAM-1(ng/mL)	TNF-α(pg/mL)
Sham	29.70 ± 2.68	26.00 ± 4.28	209.96 ± 18.77
I/R	54.04 ± 4.10*	57.30 ± 7.19*	517.13 ± 34.51*
I/R + RIPC-Exo	42.49 ± 3.05*^#^	40.83 ± 3.58*^#^	399.85 ± 27.41*^#^
RIPC-Exo + GA	51.86 ± 2.91*^&^	53.07 ± 6.80*^&^	502.65 ± 23.57*^&^

Results are represented by $\overline{x}\pm \mathrm{s }$. *Ρ < 0.05 versus Sham group; ^#^*Ρ* < 0.05 versus I/R group; ^&^*Ρ* < 0.05 versus I/R + RIPC-Exo group; n = 5 for each group

#### Table 2

(See Table 2).

#### Figure 5

(See Fig. 5).

### Effect of HSP90 in RIPC-Exo on the expression of Bax and Bcl-2

**Fig. 6:**
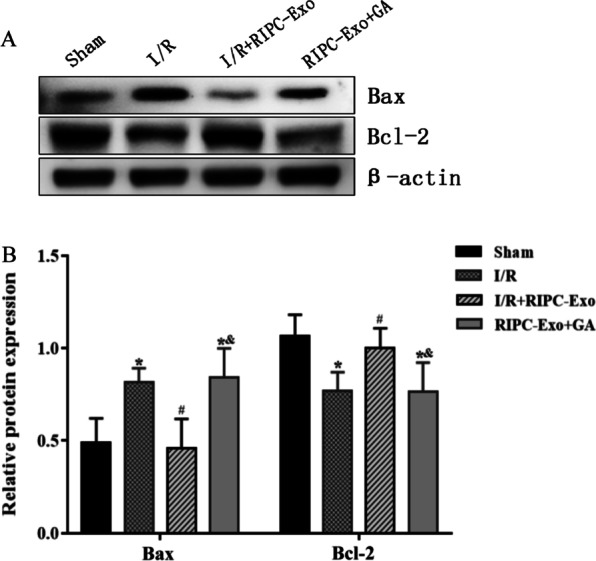
Effects of RIPC-Exo and GA on Bax and Bcl-2 proteins expression. **A** Representative Western blots showing the expression of Bax and Bcl-2 proteins. **B** The bar graph represented the relative levels of apoptosis protein expression. Dates are represented by $\overline{x}\pm \mathrm{s }$. *Ρ < 0.05 versus Sham group; ^#^*Ρ* < 0.05 versus I/R group; ^&^*Ρ* < 0.05 versus I/R + RIPC-Exo group; n = 5 for each group

To better understand the involvement of HSP90 in RIPC-Exo apoptosis, we assessed the expression of Bax and Bcl-2 proteins associated with apoptosis. Figure 6 shows that the expression levels of the Bax protein increased dramatically in the I/R group compared with the Sham group (*Ρ* < 0.05), whereas the expression levels of the Bcl-2 protein decreased significantly (*Ρ* < 0.05). In addition, the I/R + RIPC-Exo group had lower expression levels of the Bax protein and higher expression levels of the Bcl-2 protein than the I/R and RIPC-Exo + GA groups (*Ρ* < 0.05). These data suggest that HSP90 in RIPC-Exo inhibits apoptosis by decreasing Bax expression and simultaneously raising Bcl-2 expression.

#### Figure 6

(See Fig. 6).

### HSP90 in RIPC-Exo and regulates complement signalling

**Fig. 7:**
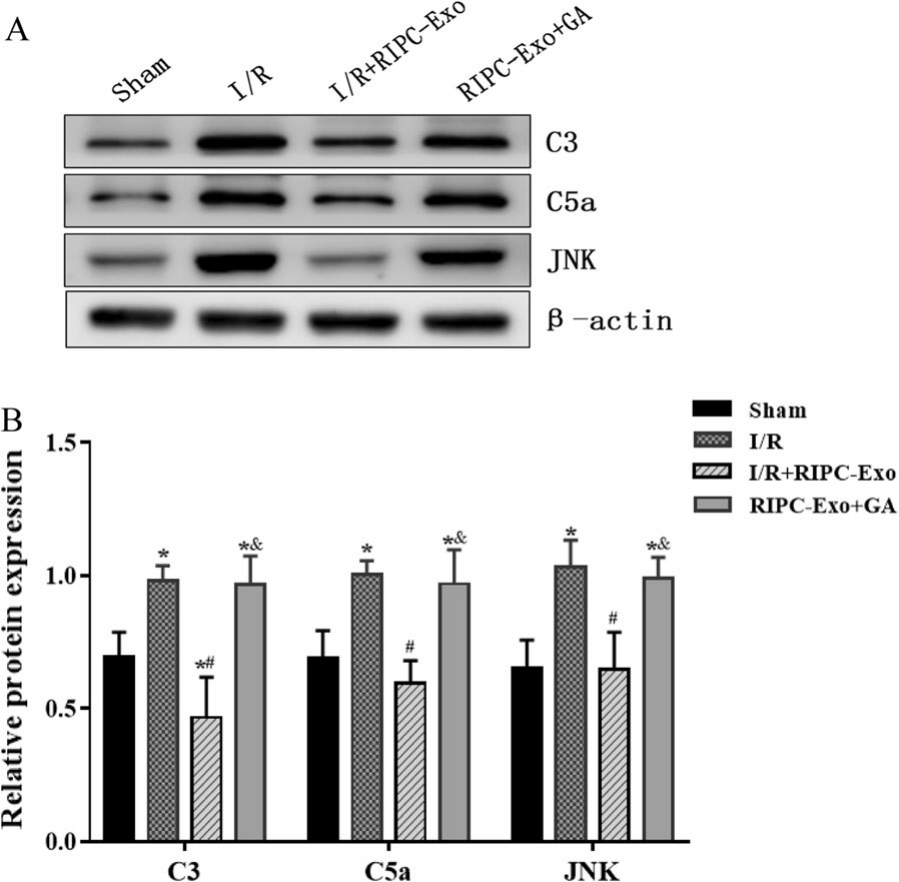
Effects of RIPC-Exo and GA on C3, C5a and JNK proteins expression**.** **A** Western blot was used to measure the protein expression levels of the C3, C5a/JNK signalling pathway in various groups. **B** The bar graph represented the relative levels of C3, C5a and JNK protein expression. Dates are represented by $\overline{x}\pm \mathrm{s }$. *Ρ < 0.05 versus Sham group; ^#^ *Ρ* < 0.05 versus I/R group; ^&^*Ρ* < 0.05 versus I/R + RIPC-Exo group; n = 5 for each group

Previous studies have confirmed that HSP90 may play a role in cardioprotection by suppressing the activation of the complement system and the JNK pathway. C3, C5a and JNK expressions were analysed to determine whether HSP90 in RIPC-Exo plays a role in complement system regulation. In this work, we discovered that the sham group had decreased expression of C3, C5a and JNK, whereas the I/R group had substantial expression (*Ρ* < 0.05, Fig. 7). Moreover, C3, C5a and JNK were dramatically downregulated in I/R rats treated with RIPC-Exo and increased after treatment with GA (*Ρ* < 0.05), indicating that HSP90 in RIPC-Exo affects cardioprotection by suppressing C3 and C5a activation and JNK signalling.

#### Figure 7

(See Fig. 7).

## Discussion

In the present study, the protective effect of RIPC-Exo on myocardial I/R injury in rats was investigated. Our data showed that RIPC-Exo treatment significantly reduced I/R-induced cardiomyocyte apoptosis, the release of myocardial markers (LDH, cTnI and CK-MB), infarct size and the serum levels of inflammatory factors (IL-1β, TNF-α and ICAM-1). Meanwhile, the activation of complement products C3 and C5a, as well as JNK signalling, was significantly inhibited with RIPC-Exo treatment. Moreover, the expression of HSP90 in the exosomes from rat plasma was significantly increased after RIPC, suggesting that HSP90 in RIPC-Exo potentially played a central role in cardioprotection by reducing cardiomyocyte apoptosis and myocardial infarction through inhibition of complement activation, local and systemic inflammation, and JNK signalling. Taken together, our results provide a novel strategy for the treatment of I/R injury.

Currently, the treatment of I/R injury still challenges cardiologists. RIPC is an inexpensive technique that could be performed in the clinical reperfusion setting by transient ischemia of an upper or lower limb, which displays strong protective effects against myocardial I/R injury [32, 33]. Furthermore, recent clinical RIPC trials have produced promising results [34, 35]. However, the underlying mechanisms of RIPC-related cardioprotection are not precisely understood. Increasing evidence suggests that RIPC may exhibit a protective effect on target organs via facilitating the secretion of microRNAs, HSP, transcription factors and cytokines, which are packed in the form of exosomes [36–38].

Exosomes, acting as signal carriers, play a vital role in cellular communication through the exchange of proteins or microRNAs between cells. Relevant studies have shown that exosomes can circulate throughout the body and exert great therapeutic potential for myocardial injury and cardiovascular disease [9, 10, 16]. Recently, accumulating data suggest that exosomes are responsible for transferring RIPC signals that elicit cardioprotection. Giricz et al. showed for the first time that exosomes isolated from hearts played a protective role in myocardial I/R injury by the transmission of RIPC signal [7]. Moreover, Li et al. [8] showed that exosomes released from RIPC plasma were crucial in mediating cardioprotective effects through transferring miR-126a-3p.

In this study, we isolated exosomes from the blood of RIPC rats and demonstrated that RIPC-Exo significantly decreased cardiomyocyte apoptosis and infarct size, promoted Bcl-2 expression and reduced the protein levels of Bax. Consistent with previous findings, our results confirmed that exosomes, as mediators of the RIPC signal, played an important role in RIPC cardioprotection. However, the specific components in the exosomes contributing to RIPC cardioprotection need to be investigated further.

To expound on the mechanism underpinning the protective effect of exosomes, we extracted exosomes from the RIPC plasma and studied the expression of HSP90, which has previously been shown to be involved in preconditioning and postconditioning cardioprotection [19, 22, 24]. Interestingly, we found that the protein expression of HSP90 in exosomes was significantly increased, and the overexpression of HSP90 greatly reduced I/R-induced cardiomyocyte apoptosis, infarct size, the release of myocardial markers and local and systemic inflammation. However, these cardioprotective effects of RIPC-Exo were significantly eliminated by HSP90 inhibitor treatment in vivo, indicating that HSP90 in exosomes isolated from RIPC was important in mediating the cardioprotective effects during I/R.

HSP90 is an intracellular chaperone that mainly exists in the myocardium as well as in the serum [18]. Being activated in response to heat stress and ischemia damage, HSP90 plays an indispensable role in homeostasis and exhibits powerful protection against cell apoptosis and oxidative stress. It is well known that HSP90 is secreted from cells by exosomes [12]. HSP90 in human embryonic stem cell-derived extracellular vesicles (exosomes) was shown to alleviate retinal degeneration by promoting retinal Müller cell retrodifferentiation [39]. Although increasing evidence suggests that HSP90 participates in cardioprotection against I/R, no study has addressed the function of HSP90 in exosomes induced by RIPC. The present study showed for the first time that exosomal HSP90 induced by RIPC may act as a carrier of the cardioprotective factor, suggesting a possible mechanism for HSP90’s involvement in RIPC protection.

The underlying mechanisms involved in the RIPC protection of exosomal HSP90 against I/R injury remain elusive. It is well known that the activation of the complement system and subsequent inflammatory responses are important features of myocardial I/R injury [25, 27, 40]. On the one hand, the excessive release of C3 and C5a during I/R are worsening factors for myocardial necrosis. On the other hand, the C3 and C5a can trigger the JNK signalling pathways to encourage the expression of inflammatory factors, including TNF-α and IL-1β, leading to myocardial inflammatory damage [28, 29].

We have previously reported that HSP90 mediated postconditioning cardioprotection through inhibition of the complement system, JNK signalling and inflammatory responses [21, 22, 24]. In the present study, we tested whether RIPC has the same effect as postconditioning in inhibiting complement activation and cardiac inflammation and whether exosomal HSP90 mediates these effects. We found that the levels of C3 and C5a, as well as inflammatory factors including IL-1β, TNF-α and ICAM-1, were higher in the I/R group and lower in the I/R + RIPC-Exo group. However, these inhibitory effects of RIPC-Exo were significantly prevented by the suppression of HSP90 with the selective HSP90 inhibitor GA, indicating that HSP90 in RIPC-Exo greatly contributed to the inhibition of the complement components C3 and C5a, as well as local and systemic inflammation during myocardial I/R. To the best of our knowledge, the effect of RIPC-Exo on the complement system has not been reported. The present study cemented the central role of complement factors in RIPC-Exo cardioprotection.

Geldanamycin is known as a potent HSP90-specific inhibitor which inhibits the chaperone function of HSP90 through suppressing the ATPase activity [41, 42]. Recently, the effect of GA and GA’ derivatives (17AAG or 17DMAG) on the regulation of HSP90 expression at the mRNA and the protein levels was reported. Chen et al. [43] found that GA selectively inhibits HSP90 protein expression, leading to the degradation of its client protein receptor-interacting protein 1 (RIP1). Furthermore, Mellatyar and Akbarzadeh’s groups indicated that 17AAG or 17DMAG suppressed HSP90 gene expression in breast cancer and lung cancer [44, 45]. In addition, Smith et al. showed that the protein level of HSP90 was decreased when treated with either 17AAG or 17DMAG in the human melanoma cell lines. The mode of depletion of HSP90 was investigated and the possible mechanism was related to an enhanced ubiquitinylation of HSP90 and thus its accelerated degradation in the ubiquitin–proteasome system [46]. The present study showed that GA decreased HSP90 expression in cardiomyocytes, however, the underlying mechanism is currently unsettled and needs further investigations.

There may be some possible limitations in this study. First, due to methodological limitations, there is currently no perfect way to purify exosomes completely. Therefore, further research is needed to determine whether additional molecules besides exosomes, such as vesicles and trace proteins, are involved in the cardioprotective effect. Second, our data showed that exosomal HSP90 was significantly increased after RIPC, suggesting that HSP90 in RIPC-Exo played a significant role in cardioprotection against I/R injury. However, other proteins or miRNAs in exosomes that contribute to RIPC cardioprotection need further investigation. Third, given that GA is a well-known inhibitor of the HSP90 function, HSP90 activity was not examined in this study. Moreover, we used only the HSP90-specific inhibitor GA to explore the cardio-protection of exosomal HSP90, considering the nonspecific effects of GA, the over or under-expression of HSP90 with viral vectors may be used in future studies.

## Conclusion

Exosomal HSP90 induced by RIPC played a significant role in cardioprotection against I/R injury, and its function was in part linked to the inhibition of the complement system, JNK signalling and local and systemic inflammation, ultimately alleviating I/R-induced myocardial injury and apoptosis by upregulation of Bcl-2 expression and downregulation of proapoptotic Bax. The present data revealed the function and mechanism of RIPC-Exo and provided a novel strategy and potential therapeutic targets for the treatment of I/R injury.

## Supplementary Information

**Additional file 1**. Original images of western blots displayed in Fig. 1E, Fig. 2A, Fig. 6A and Fig. 7A.

## Data Availability

All data generated or analyzed during this study are included in this published article.

## Abbreviations

I/R: Ischemia/reperfusion
RIPC: Remote ischemic preconditioning
RIPC-Exo: Remote ischemic preconditioning induced plasma exosomes
HSP90: Heat shock protein 90
GA: Geldanamycin
JNK: C-Jun N-terminal kinase
C3: Complement component 3
C5a: Complement component 5a
IL-1β: Interleukin-1β
TNF-α: Tumor necrosis factor-alpha
ICAM-1: Intercellular adhesion molecule-1
LDH: Lactate dehydrogenase
cTnI: Cardiac troponin I
CK-MB: Creatine kinase isoenzymes-MB
TEM: Transmission electron microscopy
NTA: Nanoparticle tracking analysis
