# Supplementary material for: Exosomal HSP90 induced by remote ischemic preconditioning alleviates myocardial ischemia/reperfusion injury by inhibiting complement activation and inflammation
Source: BMC Cardiovasc Disord. 2023 Feb 1;23:58. doi: 10.1186/s12872-023-03043-y (PMC9890892; doi:10.1186/s12872-023-03043-y)
Supplement: Supplementary file 1 — Additional file 1. Original images of western blots displayed in Fig. 1E, Fig. 2A, Fig. 6A and Fig. 7A. [file 12872_2023_3043_MOESM1_ESM.docx]

Original Images

The original western blots of Fig. 1

CD63: (26 kDa)


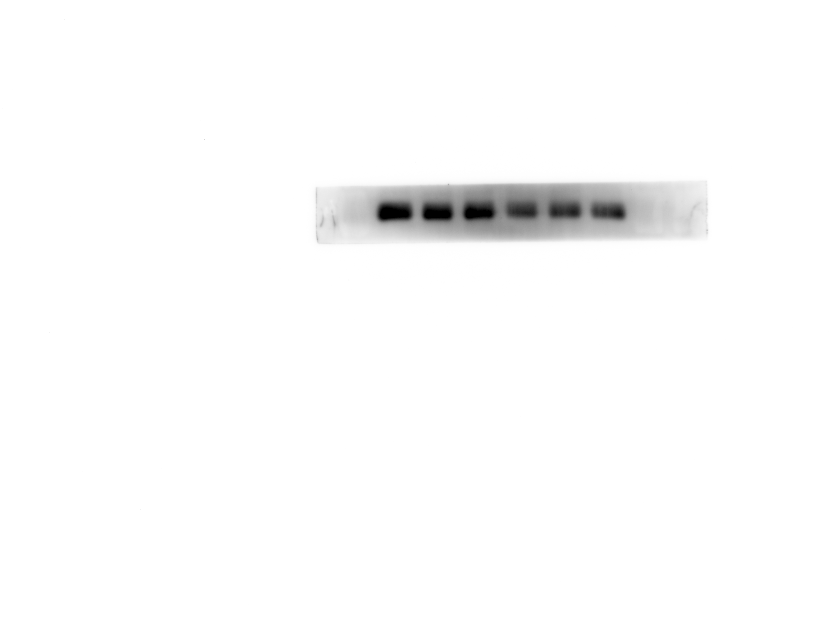


25kDa

35kDa

TSG101: ( 44 kDa)


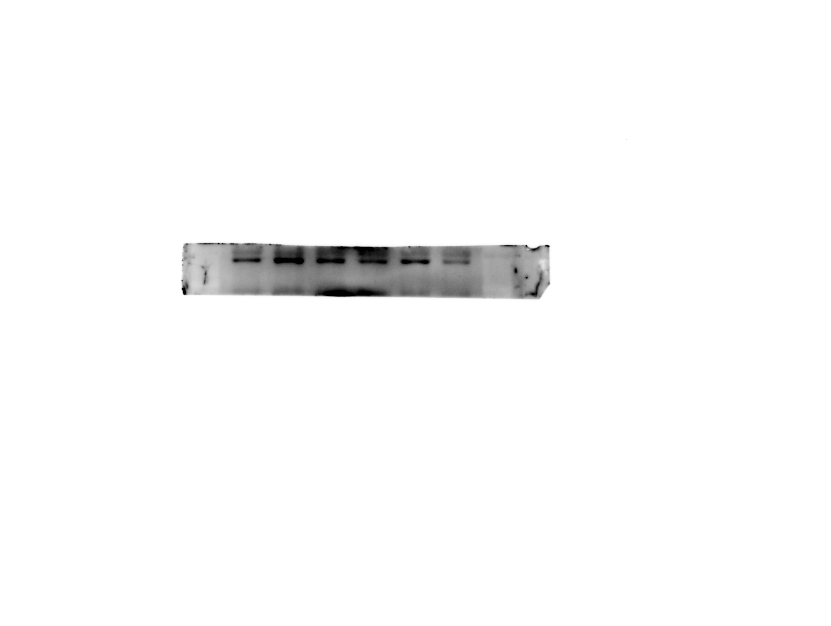


40kDa

55kDa

HSP90: (90 kDa)


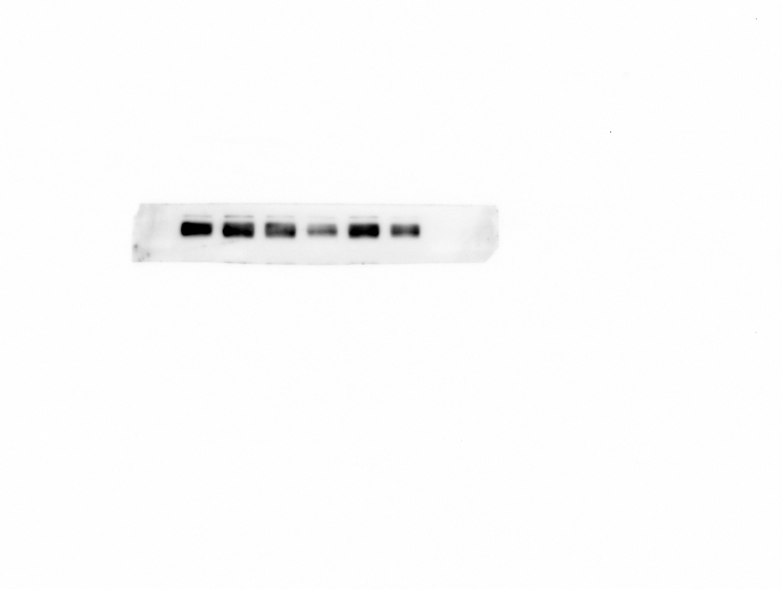


70kDa

100kDa

The original western blots of Fig. 2

HSP90:（90 kDa）


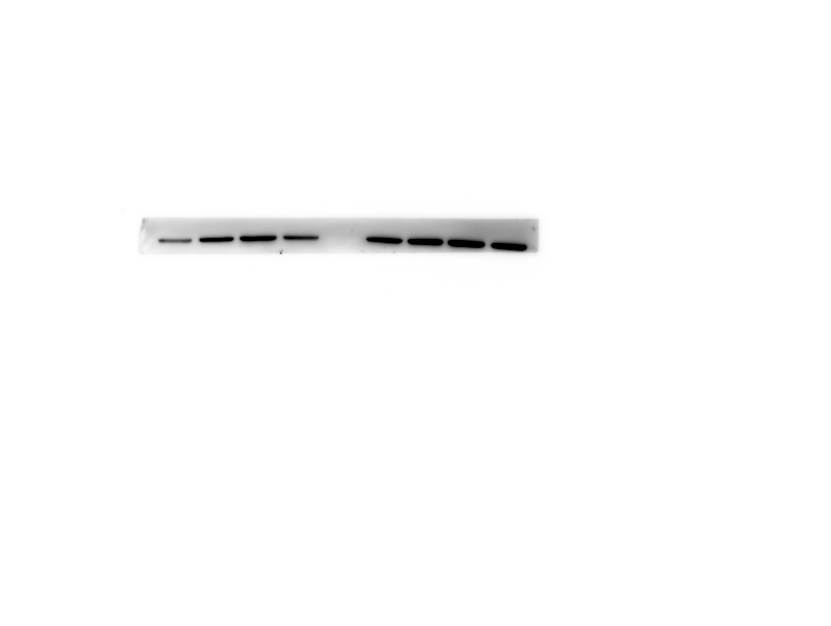


β-actin: (43 kDa)


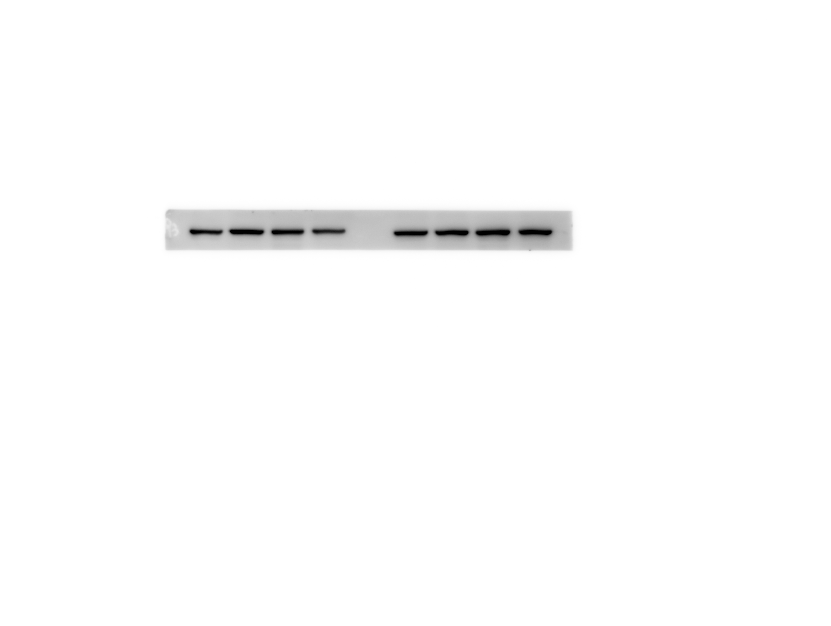


The original western blots of Fig. 6

BAX: (22 kDa)


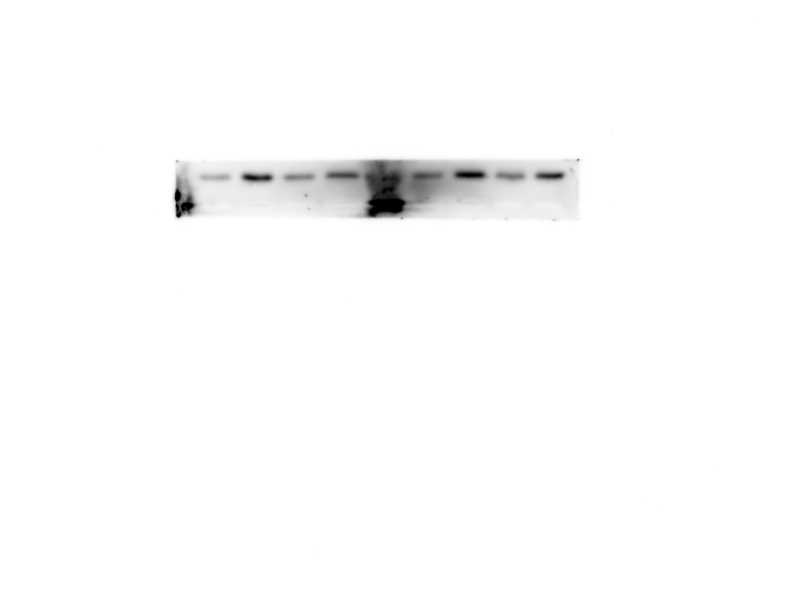


15kDa

β-actin: (43 kDa)


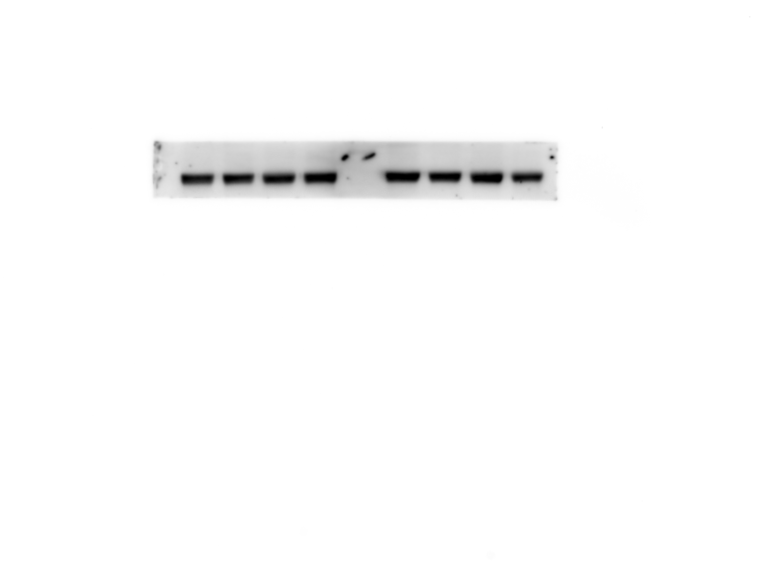


BCL-2: (26kDa)


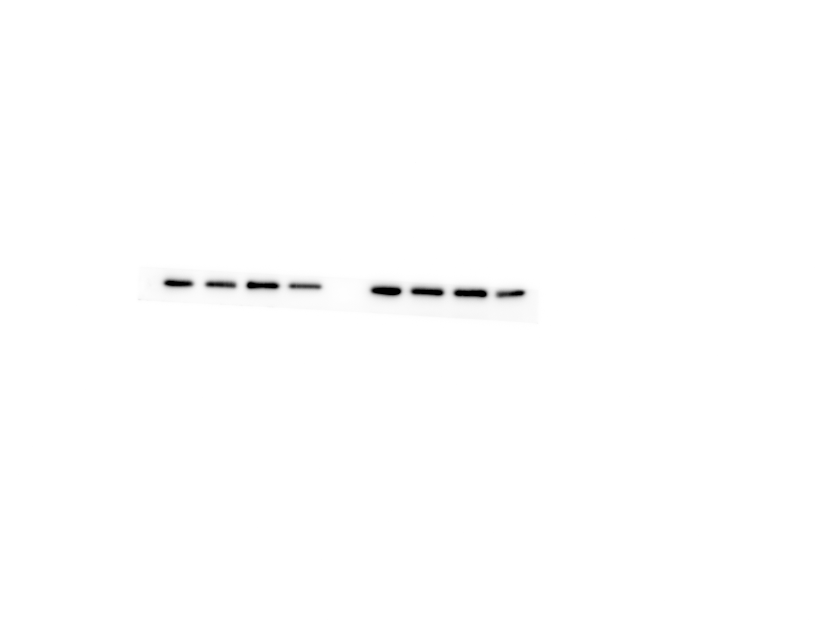


β-actin：（43kDa）


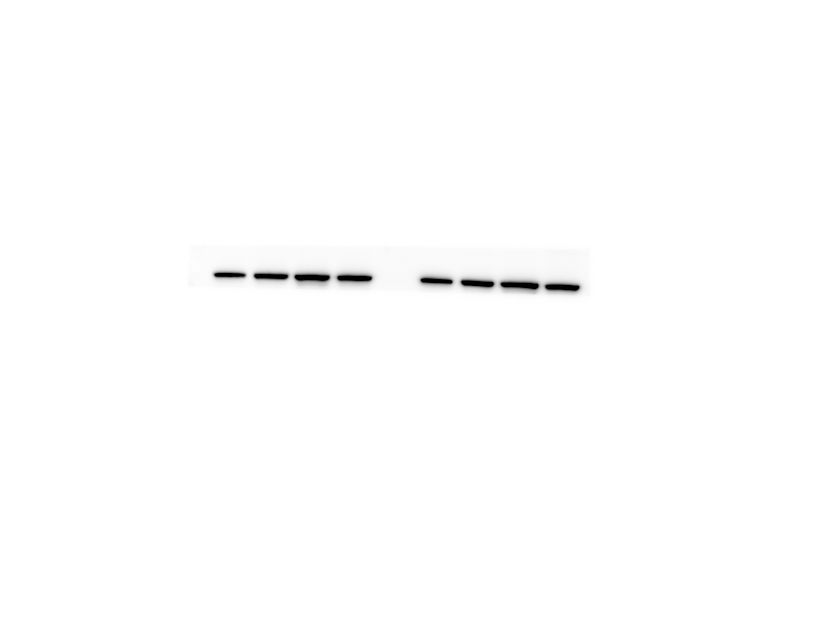


The original western blots of Fig. 7

C3: (115 kDa)


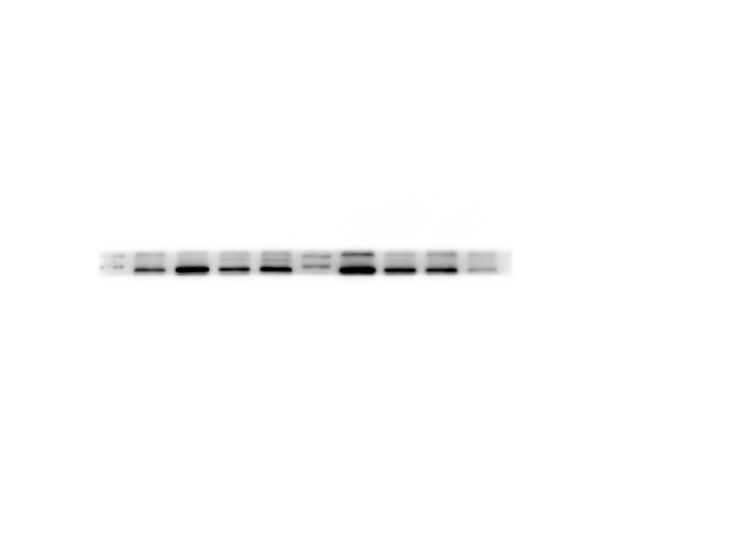


180kDa

130kDa

β-actin: (43 kDa)


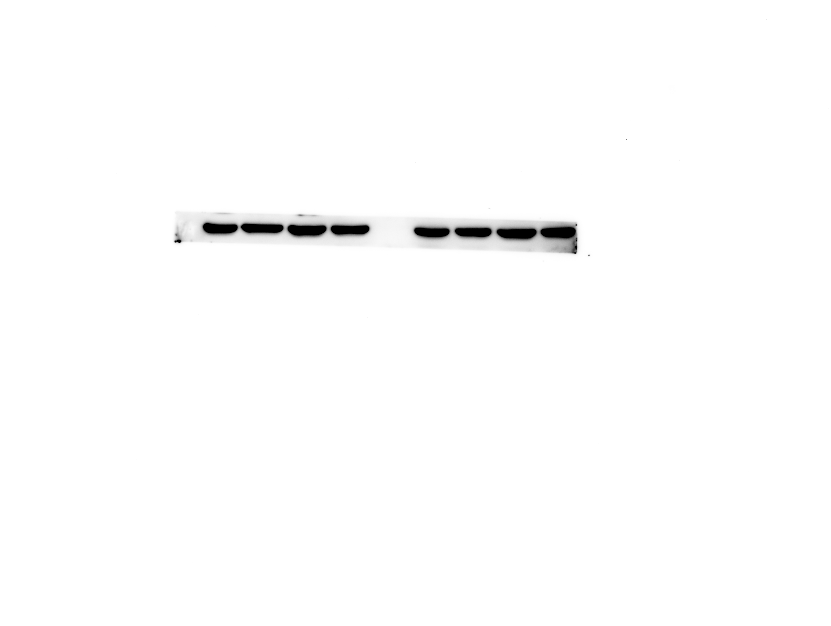


C5a:（120 kDa）


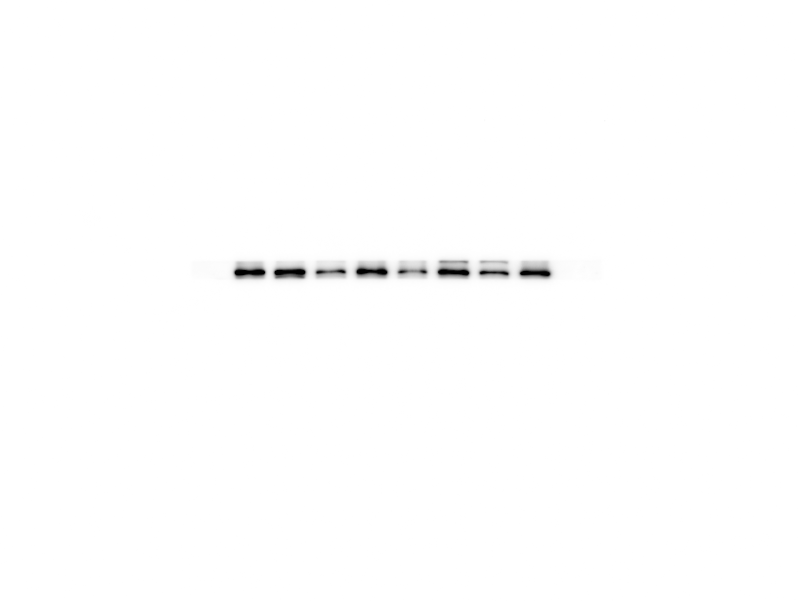


β-actin: (43 kDa)


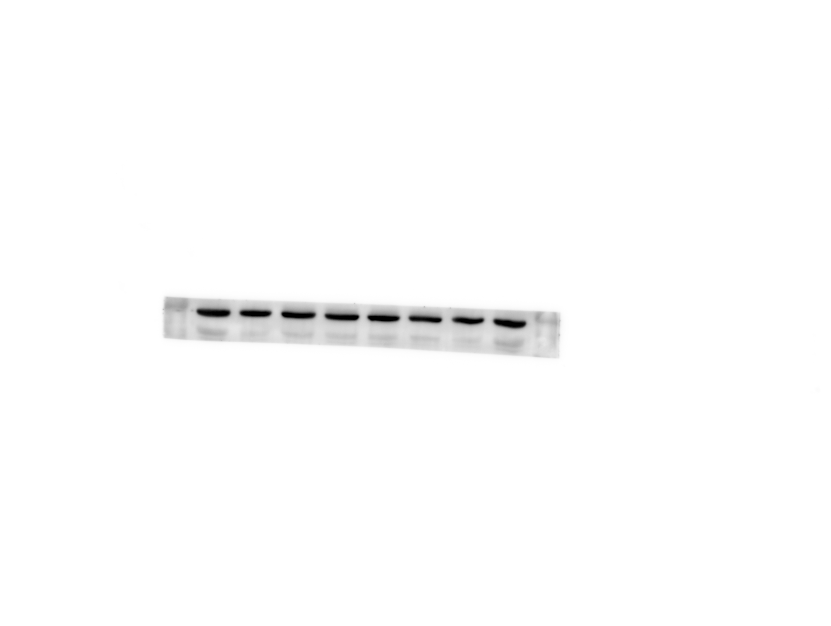


JNK: (41 kDa)


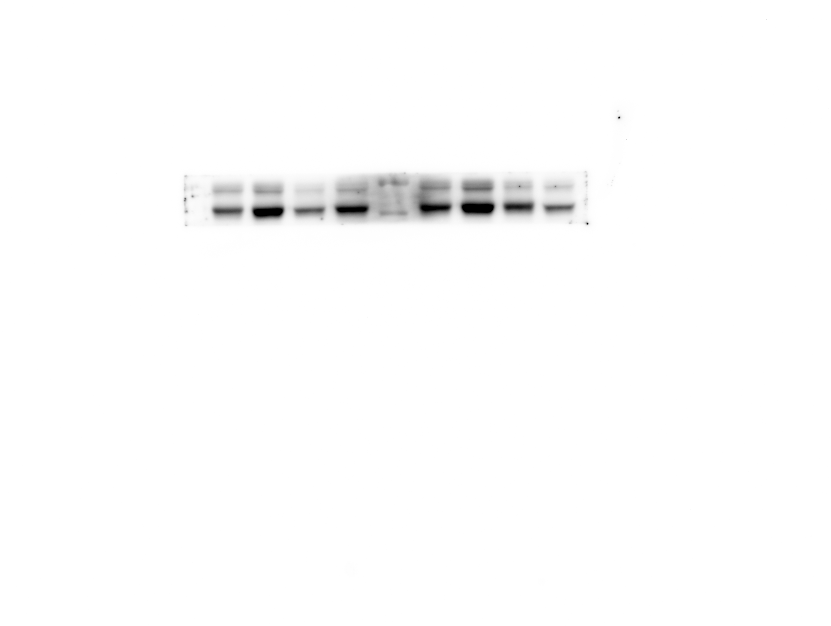


40kDa

55kDa

β-actin: (43 kDa)


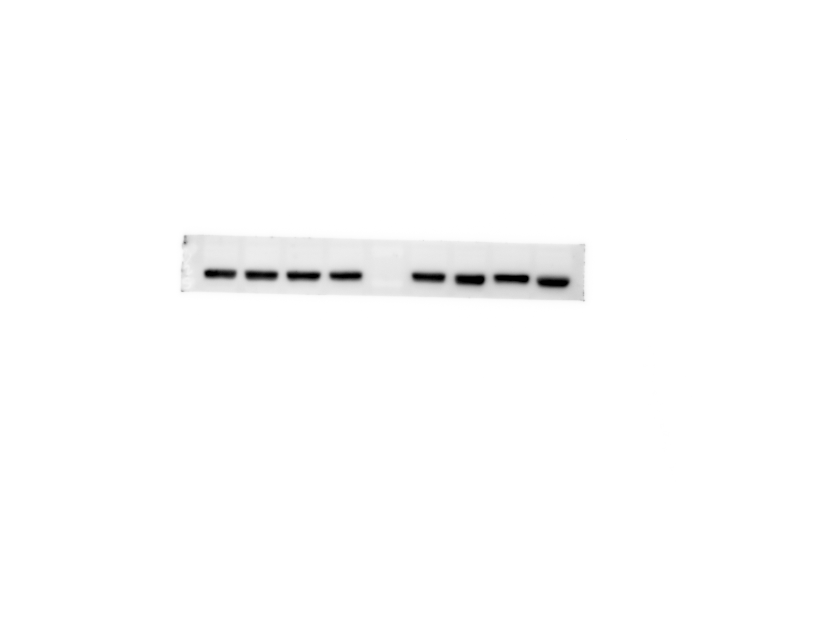


40kDa

55kDa
